# Comparative Analysis of Standard Percutaneous Nephrolithotomy (PCNL) and Mini-Percutaneous Nephrolithotomy (Mini-PCNL) for Renal Stones Larger Than 2 cm at a Rural Hospital: A Study Protocol

**DOI:** 10.7759/cureus.61963

**Published:** 2024-06-08

**Authors:** Ghanshyam Hatwar, Abhijit Dhale, Jay D Dharamshi, Ruturaj Pendkar

**Affiliations:** 1 Urology, Jawaharlal Nehru Medical College, Datta Meghe Institute of Higher Education and Research, Wardha, IND

**Keywords:** mini-percutaneous nephrolithotomy (mini-pcnl), rural hospital, stone size more than 2cm, renal stones, percutaneous nephrolithotomy (pcnl), stone-free rate

## Abstract

Background

Percutaneous nephrolithotomy (PCNL) is considered a standard treatment option for large-size renal stones but is associated with drawbacks such as bleeding and prolonged recovery. Mini-PCNL offers a less invasive alternative, but its efficacy compared to standard PCNL for renal stones larger than 2 cm remains under debate. This study aims to compare the efficacy and safety of standard PCNL versus mini-PCNL for renal stone sizes more than 2 cm.

Methods

This single-centre prospective interventional study will be conducted at Acharya Vinoba Bhave Rural Hospital (AVBRH). The study will include patients 18 to 70 years of age undergoing unilateral PCNL for renal calculi. Patients with renal stones larger than 2 cm will be enrolled. Data on stone-free rate (SFR), operative duration, hospital stay time, surgical site infection, haemoglobin (Hb) drop, need for blood transfusion, and post-surgery fever will be collected and compared between the two procedures. Statistical analysis of data will be performed using descriptive and analytical statistics.

Results

The study aims to enrol a total of 32 patients. Data analysis will be done using IBM SPSS Statistics for Windows, Version 24 (Released 2016; IBM Corp., Armonk, New York).

Conclusion

This study will provide valuable insights into the comparative outcome in terms of efficacy and safety of standard PCNL and mini-PCNL for kidney stones larger than 2 cm.

## Introduction

Percutaneous nephrolithotomy (PCNL), which typically employs a 24-30F tract, is considered the gold standard approach for treating larger renal stones owing to its higher stone-free rate (SFR) [1]. However, it comes with drawbacks and limitations such as intraoperative bleeding, blood clot formation, postoperative pain, and lengthy recovery and hospital stay duration, primarily attributed to its sizable access sheath tract [2]. As a response to these limitations, mini-percutaneous nephrolithotomy (mini-PCNL) emerged, featuring a smaller tract size ranging from 14-22F. The application of mini-PCNL dates back over two decades to the pioneering work of Jackman et al. and Helal et al. in paediatric surgery [3,4]. Despite numerous comparative studies between standard and mini-PCNL, ongoing debate centres on their respective SFR and postoperative complication rates.

Retrograde intrarenal surgery (RIRS), endoscopic combined intrarenal surgery (ECIRS), and extracorporeal shock wave lithotripsy (ESWL) appear less competitive when compared to standard PCNL in the management of kidney stones exceeding 2 cm. This raises the question of whether mini-PCNL, with its minimally invasive nature, could serve as a feasible alternative to standard PCNL in such cases.

## Materials and methods

Study objectives

The primary objective of this study is to compare SFRs between standard PCNL and mini-PCNL. The secondary objectives are to evaluate the operation time for standard PCNL versus mini-PCNL, determine hospitalization duration, assess the incidence of surgical site infections, measure the drop in haemoglobin levels post-procedure, analyze the requirement for blood transfusion, and monitor the occurrence of postoperative fever.

Study design and setting

The study design is a single-centre prospective interventional, randomized controlled study that will be conducted at Acharya Vinoba Bhave Rural Hospital (AVBRH), Wardha district, India.

Statistical analysis

We will utilize descriptive and analytical statistics to analyze the data, presenting it as mean ± standard deviation. Normality will be analyzed using the Shapiro-Wilk test. We will employ either parametric tests (such as the independent sample t-test and paired t-test) or non-parametric tests (such as the Mann-Whitney U test and Wilcoxon signed rank test) as per the distribution of data. Statistical significance will be considered when the p-value is <0.05.

Sample size

The sample size required for this study was calculated using ClinCalc (ClinCalc LLC., Chicago, Illinois) based on the outcomes of previous literature with a mean operating time in minutes. The minimum sample size calculated is 16 in each group. A total of 32 participants will be recruited.

Inclusion criteria

The study will include patients with renal stones >2 cm, patients between 18 and 70 years old, and patients scheduled for unilateral PCNL procedures.

Exclusion criteria

Patients with a body mass index (BMI) of >30 kg/m^2^, patients with active urinary tract infection and coagulopathy, and pregnant females with renal stones will be excluded.

Study method

Ethical clearance will be obtained from the Institutional Ethics Committee of Acharya Vinoba Bhave Rural Hospital (AVBRH). Preoperatively, all the patients will undergo basic investigations such as complete blood counts (CBCs), liver function test (LFT), kidney function test (KFT), PT/INR coagulation profiles, urine routine microscopy and culture, and non-contrast computed tomography (NCCT-KUB) or (CT urography). PCNL will be performed within four weeks of the scan using the standard technique (24Fr) and mini-PCNL (14Fr-20Fr). SFR, surgery time, hospitalization time, haemo­globin drop, haematocrit fall, need for blood transfusion, and post-surgery fever will be compared between the two procedures. Demographic, clinical, and operative data will be collected prospectively. This will include the patient's age, height, gender, weight, BMI, American Society of Anesthesiology (ASA) score, laterality, operation time, and hospital stay duration.

Calyceal puncture will be done using the progressive descent technique in both standard PCNL and mini-PCNL. Stone localization and tract dilatation will be performed under fluoroscopy guidance and size up to 24Fr with sequential metallic Alken dilators. The Amplatz sheath will be used to maintain the tract. The tract dilatation for mini-PCNL will be performed using a dilator size up to 14-16Fr in a single step. We will use a percutaneous universal nephroscope size 24Fr and 20Fr with a 20° angle of view (Richard Wolf GmbH™, Knittlingen, Germany) for standard PCNL and a 12Fr rigid nephroscope (Karl Storz, Tuttling­en, Germany) for mini-PCNL. Stone fragmentation will be achieved using a pneumatic lithoclast or a holmium laser. The intraoperative evaluation of renal stone and fragment clearance, and consequently the completion of the PCNL or mini-PCNL procedure, will be performed using both nephroscopic and fluoroscopic guidance. A nephrostomy tube will be used for temporary drainage as needed in both PCNL and mini-PCNL. Intravenous antibiotics will be given preoperatively to all the patients, which will be continued postoperatively for three days. Analgesics will be given postoperatively to all the patients as and when needed.

Demographic details such as age, weight, height, and gender, as well as intra- and postoperative data, will be meticulously recorded in an MS Excel (Microsoft Corporation, Redmond, Washington) spreadsheet. Complications will be evaluated using the Modified Clavien Dindo grading system for both standard PCNL and mini-PCNL procedures. On the second postoperative day (POD 2), CBC and KFT analyses will be conducted, along with a digital X-ray of the KUB region, to detect any residual stone fragments. The PCN tube will be removed if no clinically significant residual fragments are visible on the X-ray KUB or if they are clinically insignificant. Procedure success will be defined as the absence of residual fragments or the presence of clinically insignificant residual fragments (CIRF), defined as <4 mm non-obstructing, non-infectious, and asymptomatic fragments on the X-ray KUB conducted during the fourth week of postoperative follow-up. Statistical analysis will be done using IBM SPSS Statistics for Windows, Version 24 (Released 2016; IBM Corp., Armonk, New York).

## Results

The study aims to enrol a total of 32 patients. The data analysis will be conducted using the IBM SPSS software. The study protocol will adhere to ethical guidelines. Additionally, the preoperative investigations, surgical techniques, and postoperative follow-up will be standardized. This study will provide valuable insights into the comparative outcome in terms of SFR, operative duration, hospital stay time, surgical site infection, haemoglobin (Hb) drop, need for blood transfusion, and post-surgery fever between standard PCNL and mini-PNCL procedures for kidney stones larger than 2 cm.

## Discussion

Mini-PCNL, first utilized in 1997, has grown in popularity as a safer alternative for managing renal stones. Lahme advocated for its use in treating upper urinary tract stones larger than 10 mm in size. Mini-PCNL is becoming an increasingly popular modality for the management of renal stones.

The ongoing discussion about the effectiveness and safety of standard PCNL versus mini-PCNL for the management of kidney stones persists, particularly for stones larger than 2 cm [5-8]. While standard PCNL typically employs larger tract sizes (24F-30F) compared to mini-PCNL (14F-22F), the latter is not meant as a replacement for the standard PCNL technique but rather to compete with other modalities such as ECRIS, ESWL, and RIRS [9].

Research findings vary regarding SFR, with some suggesting a higher SFR with standard PCNL for stones under 2 cm but no significant difference for larger stones [10,11]. The study conducted by Cheng et al. found that mini-PCNL shows potential for achieving better SFR, especially in cases with multiple calyceal stones, potentially due to its narrower ureteroscope, facilitating access to different calyces [12]. One of the significant benefits of mini-PCNL over standard PCNL is the reduction in bleeding, which increases the likelihood of performing tubeless procedures (75%-80%) and shortens hospital stays.

Different types of lithotripsy modalities vary in their stone fragmentation ability. While laser lithotripsy has gained favour due to its efficiency compared to other lithotripsy modalities, consensus regarding the overall efficacy and incidence of complications among mini-PCNL and standard PCNL remains elusive. Surgeons must consider various factors, including tract size, stone burden, and patient factors, when determining the most suitable approach for each case.

The complication rates among mini-PCNL and standard PCNL are generally similar. In mini-PCNL, the miniaturization of endoscopic devices has certain disadvantages. The space between the nephroscope and access sheath is critical. When the diameter of the access sheath is reduced, the available space for irrigation outflow also decreases, which can result in increased renal pelvic pressure (RPP) and higher absorption of irrigation fluid. Thin sheaths can cause increased intrarenal pressure due to inadequate fluid drainage, which may extend the operation time and impair endoscopic visibility, even with minor bleeding. Additionally, these sheaths might prevent the retrieval of sufficiently sized stones for analysis and can lead to postoperative ureteric colic or urine leakage from the nephrostomy site due to small fragments blocking the ureter. Factors such as untreated preoperative urinary tract infections, high intrarenal pressure, raised perfusion pressure, longer surgery times, absorption of toxins that are released during stone fragmentation, perforation of the pelvicalyceal system, and inadequate drainage of the system post-surgery can contribute to complications.

However, mini-PCNL tends to result in longer operative times because of the necessity of breaking the calculi into smaller fragments to pass via a narrower access sheath tract [13,14]. This complexity may raise the complication risk. Furthermore, Xu et al. raised concerns regarding metabolic acidosis during mini-PCNL, which may exacerbate with prolonged irrigation time, potentially leading to more postoperative complications and slower recovery [15].

## Conclusions

This study will provide valuable insights into the comparative outcome in terms of efficacy and safety of standard PCNL and mini-PCNL for kidney stone sizes larger than 2 cm, focusing on key outcomes such as SFRs, operation time, hospitalization duration, and postoperative complications. Mini-PCNL, with its smaller tract size, potentially offers significant advantages, including reduced intraoperative bleeding and shorter hospital stays. However, it also presents challenges, such as increased intrarenal pressure and longer operative times. This study will provide a critical analysis of whether mini-PCNL can effectively mitigate the drawbacks of standard PCNL while maintaining high standards of patient care. Conducting this randomized, prospective study at Acharya Vinoba Bhave Rural Hospital will help to clarify the relative benefits and risks of mini-PCNL versus standard PCNL. The findings will contribute to the ongoing discussion about the best practices in the management of large renal stones and assist urologists in making more informed treatment decisions, ultimately enhancing patient outcomes.
